# Contextual barriers to infection prevention and control program implementation in hospitals in Latin America: a mixed methods evaluation

**DOI:** 10.1186/s13756-024-01484-4

**Published:** 2024-11-03

**Authors:** Valeria Fabre, Clara Secaira, Carolyn Herzig, Elizabeth Bancroft, Maria Paula Bernachea, Lucy Anchiraico Galarza, Bowen Aquiles, Ana Belén Arauz, Maria Del Carmen Bangher, Marisa Liliana Bernan, Sol Burokas, Alfredo Canton, Iris L. Cazali, Angel Colque, Marisabel Comas, Rosa Verónica Contreras, Wanda Cornistein, Maria Gabriela Cordoba, Silvia Mabel Correa, Gustavo Costilla Campero, Marta Isabel Chamorro Ayala, Nuria Chavez, Gabriela De Ascencao, Carlos Cruz García, Clara Esquivel, Cecilia Ezcurra, Leonardo Fabbro, Leandro Falleroni, Johana Fernandez, Sandra Ferrari, Veronica Freire, Maria Isabel Garzón, José Anel Gonzales, Lucrecia Guaymas, Fausto Guerrero-Toapanta, Diego Laplume, Sandra Lambert, César Guillermo Lemir, Paola Romina Lazarte, Itzel L. Lopez, Herberth Maldonado, Guadalupe Martínez, Diego M. Maurizi, Florencia Mesplet, Cristina Moreno Izquierdo, Gabriela Luciana Moya, Mariela Nájera, Yanina Nuccetelli, Argelis Olmedo, Belén Palacio, Florencia Pellice, Carla Lorena Raffo, Carolina Ramos, Fanny Reino, Viviana Rodriguez, Federico Romero, Juan José Romero, Graciela Sadino, Nancy Sandoval, Mariana Suarez, Maria Victoria Suayter, Maria Alejandra Ureña, Marisol Valle, Ligia Vence Reyes, Silvia Vera Amate Perez, Hugo Videla, Silvina Villamandos, Olmedo Villarreal, Maria Alejandra Viteri, Eduardo Warley, Rodolfo E. Quiros

**Affiliations:** 1 Department of Medicine, Division of Infectious Diseases, Johns Hopkins University School of Medicine, 600 N Wolfe St, Halstead 840, Baltimore, MD 21287 USA; 2 International Infection Control Program, Division of Healthcare Quality Promotion, Centers for Disease Control and Prevention, Atlanta, GA USA; 3 Clinica Conciencia, Neuquen, Argentina; 4 Clínica Universitaria Privada Reina Fabiola, Córdoba, Argentina; 5 Hospital Sociedad de Lucha Contra el Cáncer, Guayaquil, Ecuador; 6 Hospital Santo Tomas, Panama, Panama; 7 Departamento de Medicina, Universidad de Panamá, Panama, Panama; 8 Instituto de Cardiología de Corrientes “Juana Francisca Cabral”, Corrientes, Argentina; 9 Hospital Interzonal General de Agudos San Roque, Buenos Aires, Argentina; 10 Hospital Italiano de Buenos Aires, Buenos Aires, Argentina; 11 Pacifica Salud, Hospital Punta Pacifica, Panamá, Panama; 12 Hospital Roosevelt, Guatemala City, Guatemala; 13 Hospital Medico Policial Churruca Visca, Buenos Aires, Argentina; 14 Hospital El Cruce, Buenos Aires, Argentina; 15 Hospital Dr. Marcial V. Quiroga, San Juan, Argentina; 16 Hospital Universitario Austral, Buenos Aires, Argentina; 17 Hospital Municipal de Trauma Dr. Federico Abete, Malvinas Argentinas, Argentina; 18 Hospital Angel C. Padilla, Tucumán, Argentina; 19 Hospital Zonal General de Agudos Dr. Alberto Eurnekian, Buenos Aires, Argentina; 20 Hospital Regional de Zacapa, Zacapa, Guatemala; 21 Hospital Provincial de Rosario, Rosario, Argentina; 22 Hospital San Benito, Peten, Guatemala; 23 Hospital Alemán, Buenos Aires, Argentina; 24 Hospital Dr. Guillermo Rawson, San Juan, Argentina; 25 Hospital Nacional Profesor Alejandro Posadas, El Palomar, Argentina; 26 Hospital Universitario Privado de Cordoba, Cordoba, Argentina; 27 Hospital Irma de Lourdes Tzanetatos, Panama, Panama; 28 Clinical Privada Provincial, Merlo, Argentina; 29 Hospital Carlos Andrade Marín, Quito, Ecuador; 30 Hospital San Bernardo, Salta, Argentina; 31 Maternidad Nuestra Señora De Las Mercedes De Tucumán, Tucumán, Argentina; 32 Clinica Hospital San Fernando, Panama, Panama; 33 Universidad del Valle, Guatemala, Guatemala; 34 Unidad De Cirugía Cardiovascular De Guatemala, Guatemala, Guatemala; 35 Hospital Municipal de Agudos Dr. Leonidas Lucero, Bahía Blanca, Argentina; 36 Hospital Cesar Milstein, Buenos Aires, Argentina; 37 Hospital Metropolitano, Quito, Ecuador; 38 Instituto de Diagnostico, La Plata, Argentina; 39 Sanatorio Allende Nueva Córdoba, Córdoba, Argentina; 40 Hospital Vozandes, Quito, Ecuador; 41 The Panama Clinic, Panama, Panama; 42 Sanatorio Las Lomas, Av. Diego Carman 555, San Isidro, Provincia de Buenos Aires B1642 Argentina

**Keywords:** Infection prevention, Latin America, Antimicrobial resistance

## Abstract

**Background:**

Infection prevention and control (IPC) programs are essential to prevent and control the spread of multidrug-resistant organisms in healthcare facilities (HCFs). The current implementation of these programs in Latin America remains largely unknown.

**Methods:**

We conducted a mixed-methods evaluation of IPC program implementation in HCFs from Guatemala, Panama, Ecuador, and Argentina, March-July 2022. We used the World Health Organization (WHO) IPC Assessment Framework (IPCAF) survey, a previously validated structured questionnaire with an associated scoring system that evaluates the eight core components of IPC (IPC program; IPC guidelines; IPC education and training; healthcare-associated infection [HAI] surveillance; multimodal strategies; monitoring and audit of IPC practices and feedback; workload, staffing, and bed occupancy; and the built environment and materials and equipment for IPC). Each section generates a score 0–100. According to the final score, the HCF IPC program implementation is categorized into four levels: inadequate (0–200), basic (201–400), intermediate (401–600), or advanced (601–800). Additionally, we conducted semi-structured interviews among IPC personnel and microbiologists using the Systems Engineering Initiative for Patient Safety model to evaluate barriers and facilitators for IPC program implementation. We performed directed content analysis of interview transcripts to identify themes that focused on barriers and facilitators of IPC program implementation which are summarized descriptively.

**Results:**

Thirty-seven HCFs (15 for-profit and 22 non-profit) completed the IPCAF survey. The overall median score was 614 (IQR 569, 693) which corresponded to an “advanced” level of IPC implementation (32% [7/22] non-profit vs. 93% [14/15] for-profit HCFs in this category). The lowest scores were in workload, staffing and bed occupancy followed by IPC training and multimodal strategies. Forty individuals from 16 HCFs were interviewed. They perceived inadequate staffing and technical resources, limited leadership support, and cultural determinants as major barriers to effective IPC guideline implementation, while external accreditation and technical support from public health authorities were perceived as facilitators.

**Conclusions:**

Efforts to strengthen IPC activities in Latin American HCFs should focus on improving support from hospital leadership and public health authorities to ensure better resource allocation, promoting safety culture, and improving training in quality improvement.

**Supplementary Information:**

The online version contains supplementary material available at 10.1186/s13756-024-01484-4.

## Background

Healthcare associated infections (HAIs) are preventable with effective implementation of infection prevention and control (IPC) programs [1]. According to a recent qualitative study involving IPC experts from low-resource settings, suboptimal IPC program implementation is multifactorial including insufficient human, financial and technical resources, poor hospital infrastructure, and lack of or limited implementation of national guidelines [2]. The World Health Organization (WHO) has outlined resources and activities for effective IPC programs in healthcare facilities (HCFs) which are summarized in eight core components: (1) IPC program; (2) IPC guidelines; (3) IPC education and training; (4) HAI surveillance; (5) multimodal strategies; (6) monitoring and audit of IPC practices and feedback; (7) workload, staffing, and bed occupancy; and (8) built environment, and materials and equipment for IPC [3]. The IPC Assessment Framework (IPCAF) is a standardized survey that has been used to evaluate IPC program implementation in both high- and low-resource settings [4, 5]. Two recent multicenter studies, one conducted in Colombia and another one in multiple global regions, found that IPC program development was associated with country income and type of hospital (private hospitals had higher IPCAF scores than public hospitals) [4, 6]. It has been estimated that 30–50% of the population in Latin America rely on public healthcare [7]. While the IPCAF survey can identify gaps and strengths of an IPC program, it does not provide insights into socio-cultural determinants that may affect IPC implementation or the context and limitations to implementation [8, 9]. Furthermore, while the IPCAF scores correlate with other IPC indicators (e.g., the Hand Hygiene Self-Assessment Framework), it has not been validated with outcomes data. Therefore, the qualitative data we collected in this study during interviews with IPC teams becomes critically important to better understand the results of the IPCAF survey and define key opportunities for improvement to inform ongoing regional IPC improvement efforts.

## Methods

### Evaluation design, setting and population

This mixed methods evaluation comprised an IPC program self-assessment utilizing the WHO’s IPCAF survey and semi-structured interviews with HCWs directly involved in the IPC program. Hospitals were recruited either through a regional network (PROAnet), or by referral through colleagues based on their interest to participate in this evaluation [10]. Hospitals needed to have at least one individual (nurse or physician) responsible for IPC activities to be eligible to participate.

Participating countries were discussed with the U.S. Centers for Disease Control and Prevention (CDC) partners to avoid overlap with existing CDC initiatives in the region. Similarly, national authorities in each country were made aware of the evaluation and consulted regarding any concerns. All activities were coordinated by the evaluation team. The included countries have partially implemented national IPC programs according to a 2023 WHO self-assessment survey that evaluated country progress on AMR action plan implementation [11].

Healthcare professionals directly involved in IPC programs, such as physicians, IPC nurses, and microbiologists, were targeted for recruitment for interviews. The goal was to interview individuals in these three roles from 12 to 15 hospitals. Hospitals were selected for interviews based on IPCAF score, ownership, and country. Interviewees were not financially compensated for participation.

### Infection prevention and control program self-assessment survey

The WHO IPCAF survey was adapted by the evaluation team to better assess specific items relevant to the region [12]. Four questions were modified, and one question was added. Caution was taken to not alter the total score possible for each domain (see detailed description of modifications in Supplementary Material, Table 1). The adapted questionnaire was built into a platform in PROAnet (translated to Spanish) and made available to sites for completion in March 2022 [13]. The IPCAF survey has 8 sections, and each one generates a score between 0 and 100. According to the final score (ranging from 0 to 800), IPC program implementation is categorized as inadequate (0–200), basic (201–400), intermediate (401–600), or advanced (601–800).

**Table 1:** Characteristics of participating healthcare facilities (HCFs) with their IPC Assessment Framework (IPCAF) scores

HCF characteristics	Overall *N* = 37 (%)	Argentina *N* = 24 (64.8)	Ecuador *N* = 4 (10.8)	Guatemala *N* = 4 (10.8)	Panama *N* = 5 (13.5)
HCF type					
• Non-profit • For-profit	22 (59.5) 15 (40.5)	15 (62.5) 9 (37.5)	1 (25) 3 (75.0)	4 (100) -	2 (40.0) 3 (60.0)
Median bed size (IQR) • < 110 beds • ≥ 110 to ≤ 500 beds • > 500 beds	178 (125, 263) 6 (16) 25 (68) 6 (16)	182 (139, 284) 2 (8.3) 15 (62.5) 7 (29.2)	136 (101, 381) 1 (35.0) 2 (50.0) 1 (25.0)	188 (101, 612) 1 (25.0) 2 (50.0) 1 (25.0)	110 (80, 180) 2 (40.0) 2 (40.0) 1 (20.0)
Academic					
• Yes • No	30 (81) 7 (19)	20 (83.3) 4 (16.7)	4 (100) -	4 (100) -	2 (40.0) 4 (60.0)
Median IPCAF score (IQR)	614 (569, 693)	611 (557, 668)	697 (642, 752)	523 (431, 570)	667 (642, 757)

IQR: interquartile range

### Semi-structured interviews

Interviews were conducted in the participants’ language (Spanish) by a medical anthropologist (CS) through videoconference between March-July 2022 after verbal consent was obtained. An interview guide was developed by the evaluation team based on known barriers to IPC implementation in resource limited settings and guided by the Systems Engineering Initiative for Patient Safety (SEIPS) framework (Supplementary material) [14]. The SEIPS framework investigates behavioral and systematic components of healthcare practices and has been previously used to identify barriers to and facilitators of IPC and antimicrobial stewardship program implementation [15, 16]. Briefly, this framework includes three components: a work system (interacting elements that together produce performance and include people, tools, tasks, environment), a work process (i.e., how the work is done), and a work outcome. Questions were asked in an open-ended manner; prompts were only given when the interviewer deemed that they were required to encourage the conversation. Interviews lasted between 45 and 60 min and were recorded with participants’ permission for later transcription and analysis.

### Analytic approach

IPCAF survey results were analyzed using descriptive statistics. Differences between medians were calculated using the Wilcoxon Rank Sum test using STATA version 16.1 (StataCorp, College Station, TX). A 2-sided *P* value < 0·05 was considered statistically significant.

Interview data was independently coded by two investigators (VF, CS, or REQ). Directed content analysis of the interview transcripts was performed to identify themes focusing on barriers to, and facilitators of, implementation of IPC programs, and were mapped to components of the SEIPS model. IPCAF survey results and interview data were integrated and are presented in the Results section using the SEIPS framework.

## Results

### Participant characteristics and IPCAF scores

**Fig. 1:**
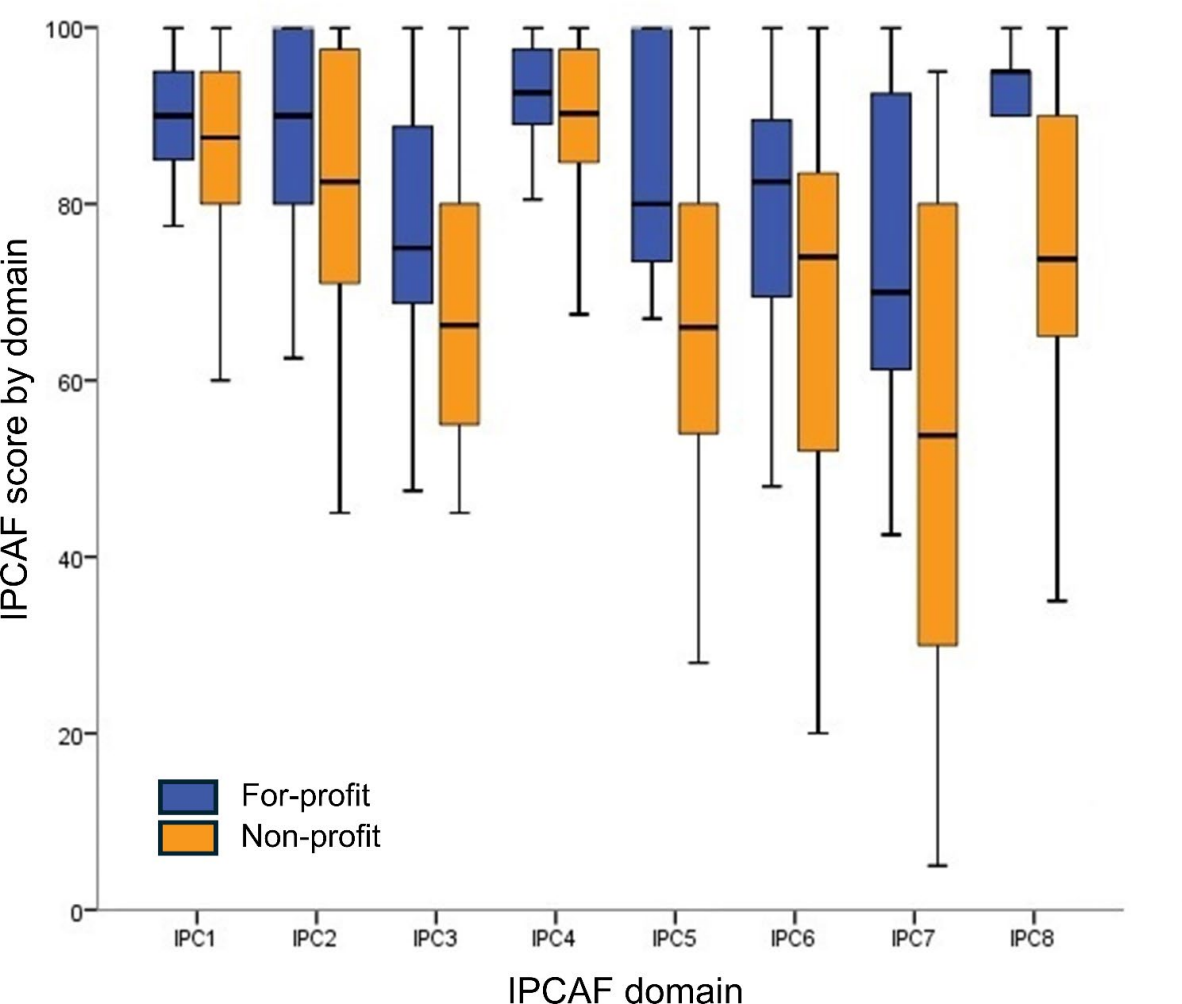
Infection prevention and control assessment framework (IPCAF) score by component stratified by healthcare facility ownership (for-profit in blue and non-profit in orange). Each IPCAF domain is scored out of a possible 100 points. Footnote: IPC1: IPC program; IPC2: IPC guidelines; IPC3: IPC education and training; IPC4: HAI surveillance; IPC5: multimodal strategies; IPC6: monitoring and audit of IPC practices and feedback; IPC7: workload, staffing, and bed occupancy; and IPC8: the built environment, and materials and equipment for IPC

Thirty-seven HCFs completed the IPCAF survey including 22 (59·5%) non-profit and 15 (40·5%) for-profit HCFs (Table 1). The overall median score was 614 (IQR 569, 693) which corresponded to “advanced” level of IPC implementation. Non-profit HCFs were less likely to score in the “advanced” category compared to for-profit HCFs (32% vs. 93%, *P* = 0.001). The lowest scores on the IPCAF survey corresponded to IPC domain 7 which addresses workload, staffing, and bed occupancy, followed by the domains assessing IPC education and training (IPC domain 3) and multimodal strategies (IPC domain 5). The median scores for these three domains were 62·5 [IQR 45, 90]; 70 [IQR 60, 82·5]; and 75 [IQR 62, 95], respectively (Fig.  1). The highest score corresponded to IPC domain 4 which evaluates HAI surveillance (median 90·6 [IQR 87, 97]). Compared to for-profit HCFs, non-profit HCFs had significantly lower scores in multimodal strategies (median score 66 [54, 80] vs. 80 [IQR 71, 100], *P* = 0·013); workload, staffing, and bed occupancy (median score 54 [IQR 30, 80] vs. 70 [IQR 57, 92], *P* = 0.01); and built environment, material, and equipment for IPC (median score 74 [IQR65, 90] vs. 95 [IQR 90, 95], *P* = 0·003). Answers to each IPCAF question are shown in Suppl. Table 2.

### Semi-structured interviews

To better understand the context of survey results, we interviewed 40 individuals from all four countries including 13 (32%) physicians and 16 (39%) nurse members of the IPC program, and 11 (27%) microbiologists from four for-profit (all with IPCAF scores in the “advanced” category”) and 12 non-profit HCFs. Non-profit HCFs that participated in interviews had IPCAF scores in “basic” [*n* = 1], “intermediate” [*n* = 7], and “advanced” [*n* = 4] categories). Perceived barriers to effective IPC program implementation are summarized in Table 2. Facilitators to IPC program implementation are summarized in Table 3. We integrated interview data with IPCAF survey results using the SEIPS framework to identify gaps in IPC implementation, barriers, and facilitators which are discussed below.

**Table 2:** Perceived barriers to effective infection prevention and control (IPC) program implementation by IPC personnel (physicians and nurses), and microbiologists

	Barrier and exemplar quotation
Organization	• Limited and/or non-sustainable hospital leadership support towards IPC *“More than a year ago*,* we developed an IPC plan and presented it to hospital leadership for approval*,* but we have still not gotten an answer” -IPC physician-* *“They [hospital leadership] don’t do anything with the data we share” -IPC physician-* • Suboptimal communication with hospital leadership *“We don’t have fluent communication with hospital leadership. For example. We have to mail requests to the hospital director*,* and wait for weeks or months to get a response” -IPC physician-* • Hospital bureaucracy to appoint new personnel, approve purchases, etc. • IPC physicians lack protected time for IPC activities • Inconsistent unit leadership support for IPC activities • Lack of patient safety culture *“This [patient safety culture] is something we have to get better at. Many [HCWs] remember to protect themselves but forget about the patient. Our hand hygiene compliance is always high for moments after interacting with the patients but always low for moments before touching the patient” -IPC nurse-* • Suboptimal compensation of HCWs *“Many of us have multiple jobs to make a decent living” -IPC physician-* • Suboptimal work climate, low morale • Insufficient human resources and workload across disciplines (including in IPC) *“We are always trying to put out the fire*,* we don’t have time to really think what are the priorities and how to approach them” -IPC nurse-* • Frequent staff turn-over • Power distance “*Our society favors men*,* and there is a hierarchical structure that does not help us” -IPC nurse*- • Lack of unit accountability for low performance
Healthcare personnel	• Limited HCW’s awareness of the importance of IPC programs • Limited HCW’s IPC knowledge • HCW resistance to feedback and/or change • Limited HCW motivation to change • EVC services staffed by external companies (makes communication with IPC a bit more challenging)
Tasks	• Unable to reach all relevant groups/areas (due to inadequate IPC staffing) • Difficulty in translating data into action (majority are familiar with measuring and reporting but less familiar with implementing changes) • Do not measure impact of implemented interventions • IPC teams are responsible for a broad range of issues • Limited testing availability for *Clostridioides difficile*
Tools and technology	• Limited technical resources *“We have to coordinate who is going to use the computer because we only have one” -IPC nurse-* *“Sometimes*,* a medical student or a resident gives us their old computer. When they die*,* we have to bring our own” -IPC nurse-* • Limited training opportunities for the IPC team *“The [hospital leadership] asked us to do it on weekends because there is less clinical work than on weekdays” -IPC nurse-* *“We bought with our own money the supplies we needed for the hand hygiene workshop” -IPC nurse-* • Limited training opportunities for HCWs • Limited supplies *“We can’t provide feedback on hand hygiene compliance when we haven’t been able to provide towels to dry their hands” -IPC nurse”* • Lack of efficient methods to perform HAI surveillance • Lack of support to process, analyze, or display HAI data • Fragmented electronic health records
Physical environment	• Limited space • The microbiology laboratory has limited operating hours • Hospital infrastructure
External environment	• COVID-19 pandemic *“The pandemic stopped our normal work. We have to go back to measuring hand hygiene compliance and other basic IPC activities” -IPC physician-* • Lack of incentives to reduce HAIs • Limited support from public health authorities for IPC implementation “*IPC programs should be a public health priority. We need their support to make IPC programs a hospital standard”*

HCW: healthcare worker, HH: hand hygiene, HAIs: healthcare acquired infections

**Table 3:** Perceived facilitators of effective infection prevention and control (IPC) program implementation by IPC physicians and nurses, and microbiologists

	Facilitator and exemplar quotations
Organization	• Empower IPC nurses *“We let them [IPC nurses] present the data [HAI rates] and we are there to support them” -IPC physician-* • Empower patients to be IPC advocates (e.g., teaching patients how to care for surgical wounds) • Empower bedside nurses to be IPC champions *“We started offering this to nurses as a way to engage them in IPC. They rotate through our service*,* they learn about ID*,* shadow the IPC nurse… They love it…. Then they teach other in their units” -IPC physician-* • Dedicated budget • Frame IPC in the context of patient safety *“It is important for us [IPC nurses] to remind them [physicians]*,* HAIs are preventable…And that they can make a difference” -IPC nurse-* • Hospital director is an active member of the IPC committee • Multidisciplinary work • Top-down approach *“If the hospital director does not set it as an expectation*,* no one will listen to us” -IPC physician-*
Person	• Having an expert in quality in the IPC team
Tools and technology	• Competency-based training • Computers to track IPC outcomes data • IPC-specific journal club to increase IPC nurses’ confidence in addressing issues with front-line providers
Tasks	• Color code HAI rates or hand hygiene compliance (red, yellow, green) for easier visualization of data • Use of SWOT framework for effective strategic planning • Weekly check-in meeting to plan activities for the week for the members of the team • Units participate of implementation of initiatives • Frequent internal meetings • Alternate HAIs on a quarterly basis
External environment	• External hospital accreditation • COVID-19 *“The pandemic did help increase the visibility of the IPC program*,* and our value” -IPC physician-* • Interaction with public health authorities *“They [national authorities] came to visit us and left very helpful recommendations on things to we need to work on. It is good to hear what we are doing right and what we need to improve on” -IPC physician-*

#### Organization

According to the IPCAF survey, 30% (11 of 37) of HCFs had fewer than one IPC professional per 110 beds and the IPC professional did not work full time in another 24% (9 of 37). Furthermore, 70% (26 of 37) of HCFs did not maintain an adequate workload of HCWs. Interviewees overwhelmingly agreed human resources were insufficient for the usual very wide scope and responsibilities of IPC programs. IPC physicians rarely had protected time to perform IPC program activities. They perceived the following challenges to maintaining appropriate IPC staffing (especially in non-profit HCFs): delays in appointing new personnel due to bureaucracy, more attractive compensation packages in for-profit HCFs, and poor work climate. While interviewees acknowledged their interactions with hospital leaders were collegial, they were not necessarily collaborative (they did not have the support to find or implement solutions). Participants reported generally low prioritization of IPC programs by HCF directors and cited tight budgets as an impediment to allocate more resources to the IPC program. Interviewees also perceived a “top down” approach from the HCF director (where decisions are made by the director and then communicated to the staff) was the nudge needed to improve HCWs’ engagement in IPC and adherence to IPC best practices.

Interviewees perceived stronger patient safety culture was needed to improve the engagement of HCWs, unit and HCF directors in IPC. Some IPC nurses stated they were perceived as the hospital “police” rather than partners with a common goal towards improving patient care. Other activities affected by cultural barriers are discussed in *Tasks*. According to the IPCAF survey, 78% (29 of 37) of HCFs did not evaluate safety culture, yet 70% (26 of 27) had representatives of quality and patient safety in their IPC committees (100% [15 of 15] in for-profit and 50% [11 of 22] in non-profit HCFs).

#### Healthcare personnel

There were several barriers to broader HCW engagement and participation in IPC. Interviewees reported that nurses were generally more engaged in IPC than physicians; however, work overload was an impediment for more nurses to champion IPC activities in their units. This was evidenced by IPCAF results, 41% (9 of 22) of non-profit HCFs did not have staff that did not belong to IPC that could serve as trainer or help with monitoring.

Another perceived barrier to HCW compliance with IPC guidelines was HCWs’ perceptions that HAIs or multi-drug resistance organisms (MDRO) were not a problem in their HCF, and a limited knowledge on the impact of IPC on patient outcomes. According to the IPCAF survey, HCFs most commonly (57%, 21 of 37) offered non-mandatory IPC training for their HCWs upon hiring or annually, while 19% either did not offer training or training was only done upon hiring. IPC personnel shared that the main limitations to providing more training in IPC to HCWs included lack of a budget and lack of leadership support to conduct in-service training during work hours.

#### Tasks

Prevention guidelines are a basic core activity of any IPC program. According to the ICPAF survey, most (95%, 35 of 37) HCFs implemented basic IPC guidelines such as those related to hand hygiene, transmission-based precautions, disinfection, and sterilization. Fewer implemented guidelines for the prevention of surgical site infection (SSI) (78%, 29 of 37), transmission of MDROs (78%, 29 of 37), or safe injections (59%, 22 of 37). Insufficient time/personnel to develop guidelines was a common theme revealed during interviews.

Compliance with hand hygiene and central-line associated bloodstream infection prevention bundles were measured at least quarterly by 84% (31 of 37) and 78% (29 of 37) of HCFs, respectively, according to the IPCAF survey. These data were usually shared with unit directors (by 76% of HCF, 28 of 37) and less frequently with HCWs (by 54% of HCFs, 20 of 37). HAI rates were shared with unit leaders in 84% (31 of 37) of HCFs, with HCWs in 70% (26 of 37) of HCFs, and with executive leadership in 54% (20 of 37) of HCFs. During interviews, IPC nurses indicated that power distance related to gender (with women perceived as less powerful than men) and role (with those with a nursing degree perceived as less powerful than those with a medical degree) were barriers to sharing and discussing IPC process and outcome measures with other roles. In some cases, HCF leadership had concerns about disseminating these data among HCWs within the facility due to potential misuse of the information in social media in retaliation to low wages. Some teams overcame this type of situation by implementing color-coded compliance data rather than actual numbers. In one HCF, the IPC team had recently incorporated an expert in patient safety into their team who made positive changes related to dissemination and communication of IPC compliance data. Another IPC team shared that improving IPC nurses’ evidence-based knowledge through journal clubs improved their ability to communicate with physicians regarding the need to implement an IPC bundle or adhere to best IPC practices.

In the IPCAF survey, HAI surveillance had an overall high score. However, 22% (8 of 37) of HCFs did not have standardized processes in place to regularly review the quality of information collected for surveillance. IPC personnel also shared inefficiencies in their daily work related to HAI surveillance mostly due to suboptimal technical resources (e.g., lack of office space, working with very old computers that needed to be shared, and limited information and technology support). This limited HAI surveillance to the ICUs, although, interviewees recognized opportunities to work in other clinical areas such as the wards, operating theaters, etc. Sometimes, IPC personnel were expected to prioritize policy enforcing activities (e.g., fingernail policy) over other core IPC activities. Furthermore, according to the IPCAF survey, 23% (8 of 37) of HCFs had no objectives or measurable outcome indicators of the IPC program.

While most (89%, 33 of 37) HCFs reported using multimodal strategies to implement IPC guidelines including the use of bundles in the IPCAF survey, interviewees reported a desire for training in quality improvement initiatives, and that they often lacked the support to assess the success of multimodal interventions and how to address challenges. For-profit HCFs were more likely to report linking patient safety and quality colleagues to developing and promoting multimodal IPC strategies than non-profit HCFs (93% [14 of 15] vs. 50% [11 of 22]).

#### Tools and technology

Interviewees reported a range of resources needed to improve their work and to improve HCWs’ compliance with IPC best practices ranging from hand hygiene supplies to office space and computers. Although most participants indicated their HCFs had an electronic medical record (EMR), it often did not provide tools to expedite IPC work such as a list of patients, flags for patients requiring transmission-based precautions, etc. Similarly, microbiology results were frequently not integrated into the EMR which required teams to rely on the IPC nurse to communicate about patients with MDRO and implementation of isolation precautions. Interviewees thought investments in software were cost prohibitive.

#### Physical environment

According to the survey, 24% (9 of 37) of HCFs frequently placed patients in beds in hallways and 51% (19 of 37) were able to ensure a minimum distance between beds for all departments. Most for-profit (87%, 13 of 15) and fewer non-profit HCFs (55%, 12 of 22) reported reliable access to hand hygiene devices in all areas. Filtered air systems were present in 80% (12 of 15) and 36% (8 of 22) of for-profit and non- profit HCFs, respectively. Reliable, ready-to-use sterile and disinfected equipment was unavailable for 19% (7 of 37) of HCFs.

#### External influences

Interviewees perceived their interactions with subnational/national health authorities very positively. Some of these interactions included audits of the IPC program, support for identification of antimicrobial resistant bacteria, and reporting of HAIs to the national surveillance network. Some expressed the reporting system could be simplified, and they wished for more feedback regarding surveillance results and guidance on action items. They also thought it would be beneficial to receive more technical assistance with the development of high-quality evidence-based IPC education for different HCW roles. IPC personnel perceived external HCF accreditation to be an important facilitator to improve resources, secure protected time for the IPC program, and prioritize IPC activities in the HCF. According to the survey, most (78%, 29 of 37) HCFs reported relevant surveillance data to sub-national and/or national authorities such as HAIs.

## Discussion

We evaluated IPC program implementation in 37 HCFs from Guatemala, Panama, Ecuador and Argentina using a mixed-methods approach, including the IPCAF survey and semi-structured interviews with IPC personnel and microbiologists to better understand current gaps and barriers to sustainable and effective IPC programs. The largest gaps based on the IPCAF survey were in the domains of workload, staffing, and bed occupancy; education and training; and implementation of multimodal strategies. In general, gaps were more severe among non-profit HCFs. Interviewees provided important insight and context to these gaps and shared facilitators for IPC implementation. The main barriers were related to limited budgets, inadequate human and technical resources, suboptimal patient safety culture, limited knowledge of IPC, and limited hospital leadership support. A major facilitator of sustainable IPC implementation perceived by interviewees included hospital accreditation.

Education and training in IPC is essential to improve HCWs’ adherence to IPC guidelines and for IPC teams to effectively implement guidelines [6, 17, 18]. Similar to prior IPCAF surveys that evaluated IPC implementation in resource-limited settings, HCFs in this evaluation scored low on this domain [4]. Perceived barriers reported by our participants included lack of funds to cover training courses for the IPC team, lack of protected time for HCWs to receive training, and insufficient staff to provide more frequent and effective education (education was most commonly delivered via classroom instruction). In a recent survey conducted by members of the evaluation team, up to 60% of Latin American HCWs reported not having received IPC education, with physicians being more likely to report lack of training in IPC than nurses or environmental care personnel [19]. This is not surprising as IPC education in many Latin American countries has traditionally focused on nursing staff. Improving access to evidence-based literature among IPC personnel was perceived as a facilitator to promote IPC guideline implementation as it improved the teams’ confidence in discussing guidelines with medical teams. A study evaluating IPC experts’ perspective on IPC implementation from resource-limited settings reported strengthening an IPC career path and identification of local champion trainers as important to improve IPC education in hospitals [2]. Another study assessing educational resources among members of the International Society for Infectious Diseases society reported most participants wished for step-by-step instructions on how to implement IPC strategies [20]. Our participants indicated it was extremely helpful to receive visits/audits by national/subnational authorities that left them with specific recommendations for areas for improvement. They also indicated technical assistance with development of high-quality training resources by public authorities/scientific societies for HCWs in different levels of training and career stages would be highly beneficial. Other specific areas in which participants desired more training opportunities were effective communication and quality improvement implementation.

Direct support by HCF leaders has been associated with improved IPC outcomes (lower HAI rates) in high-income countries due to better compliance with IPC policies and procedures by HCWs [21, 22]. However, HCF leadership engagement and support towards IPC remained limited among our participating HCFs. This was attributed among other things to underappreciation of the importance of IPC programs, or lack of strong patient safety culture by HCF leadership. The later refers to an organizational commitment to create and promote an inter-rank/disciplinary collaboration which is essential to ensure compliance with IPC policies and procedures [23]. A recent survey evaluating patient safety culture in Latin American hospitals showed an overall low perception of patient safety culture (48% as a global measure) [24]. Removing staff hierarchy may help with communication around IPC process and outcome measures and developing strategic plans with unit or hospital directors. This is particularly important in HCFs where the IPC nurse may be the main individual responsible for the IPC program. Our participants perceived power distance as a barrier to guideline implementation; however, they also suggested a “top down” approach was necessary to improve HCWs’ engagement in IPC, similar to what has been reported in other regions that share similar socio-cultural patterns [8, 25]. These findings underscore the need for more research to better evaluate what behavioral approaches are most effective in positively influencing HCWs towards IPC in Latin America.

Another cited barrier to improving resources for the IPC program was the lack of a dedicated budget. The few studies that have evaluated cost-effectiveness of IPC programs in Latin America have shown encouraging results [26, 27]. A single-center study from Argentina reported ~ 300,000USD net savings for a 20% reduction in HAIs in a year [27]. Hospital administrators should be aware of these data and work with IPC teams in developing investment plans for IPC programs. This is another opportunity where national and/or subnational levels could help set standards and improve resources (e.g., nurse-to-patient ratio). According to the 2023 WHO self-assessment survey that evaluated country progress on AMR action plan implementation, 32% (9 of 28) of Latin American countries that took the survey had not implemented an IPC program highlighting the need to urgently prioritize IPC program implementation at the country level, and efforts to identify levers to help HCFs better support IPC activities [11].

Our evaluation has several limitations. Selection bias may have occurred as we did not use a randomized sample, and we may have included HCFs that were more engaged or had more robust IPC programs. Even though the sample included a mix of for-profit and non-profit HCFs, most scored in the “intermediate” or “advanced” level of IPC implementation. Additionally, only four of the 31 countries in Latin America were included. Furthermore, 65% of HCFs completing the ICPAF were from Argentina. Although other studies using different recruitment methodologies and in other countries in Latin America not included in the present study have provided similar results, findings from ours and other studies may be underestimating gaps in IPC implementation [4, 6]. While interviews provided unique insights into the challenges associated with IPC implementation in Latin American hospitals, we did not interview HCF directors. Future studies addressing their perspective are needed. Finally, we used the SEIPS model to develop the interview guide to ensure many relevant factors that can influence program implementation were considered. However, due to the wide scope of IPC programs, there were some areas that were not addressed in detail in our evaluation such as challenges related to complying with bed occupancy recommendations.

## Conclusions

This evaluation allowed us to identify several opportunities to improve IPC program implementation in Latin American hospitals (see a summary of future considerations to strengthen IPC in the region in the **Box**). Based on our findings, improving technical and human resources to allow IPC teams to perform their work more efficiently and be able to expand their activities to the hospital more broadly, nourishing a culture of safety, and improving resources to facilitate guideline implementation and to increase IPC knowledge for IPC staff and HCW are crucial to building robust IPC programs in the region. National or subnational authorities can play an important role in addressing many of these gaps. It is critical for countries to identify the levers that will allow HCFs to invest more resources in IPC programs. There might be additional gaps in IPC implementation that were undetected through our study. Additional evaluations in other countries in Latin America are needed.


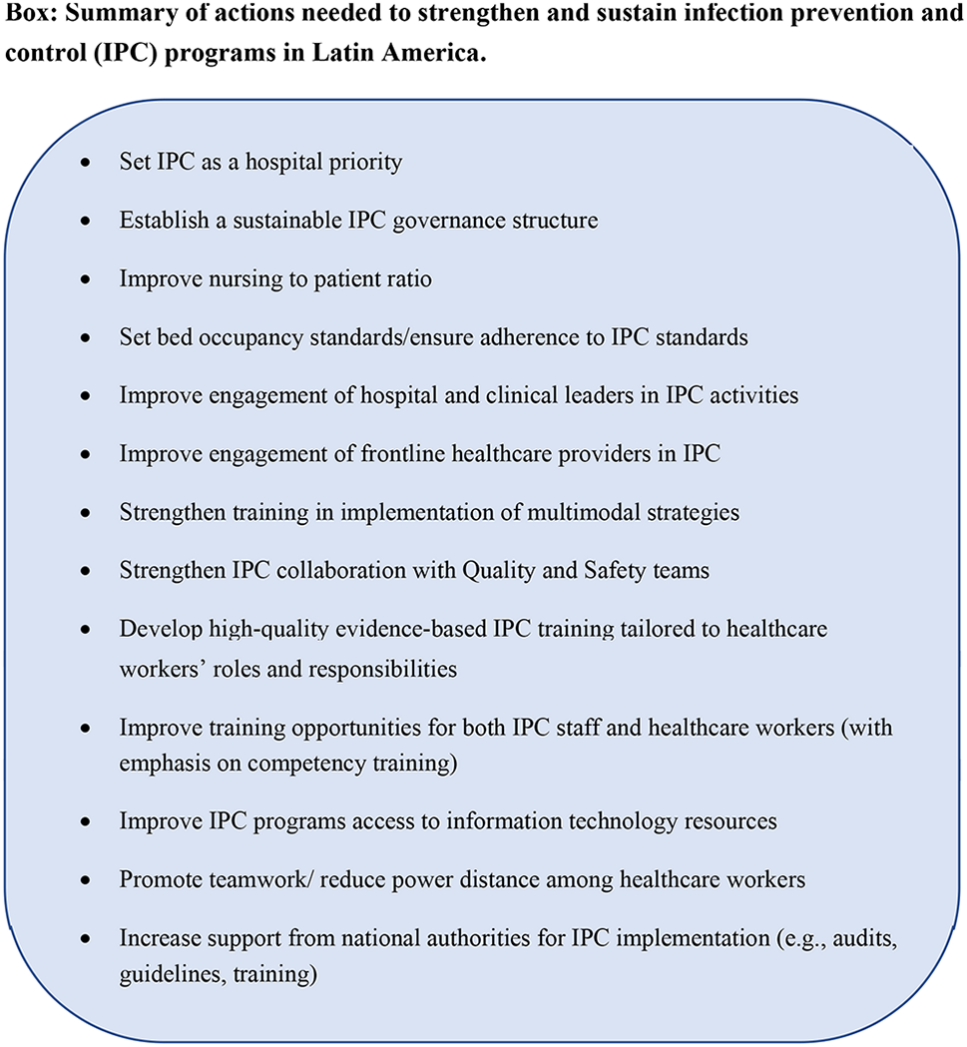


## Electronic supplementary material

Below is the link to the electronic supplementary material.

Supplementary Material 1

## Data Availability

No datasets were generated or analysed during the current study.
