# Supplementary material for: Contextual barriers to infection prevention and control program implementation in hospitals in Latin America: a mixed methods evaluation
Source: Antimicrob Resist Infect Control. 2024 Nov 3;13:132. doi: 10.1186/s13756-024-01484-4 (PMC11533356; doi:10.1186/s13756-024-01484-4)
Supplement: Supplementary file 1 — Supplementary Material 1 [file 13756_2024_1484_MOESM1_ESM.docx]

**Supplemental Appendix**

**Contextual barriers to Infection Prevention and Control program implementation in hospitals in Latin America: A Mixed-Methods Study.**

Fabre et al.

**Table of Contents**

Table S1. Adaptation of World Health Organization Infection Prevention and ControlAssessment

Framework

Table S2: Complete answers to Infection Prevention and Control Assessment Framework

(IPCAF) survey

Supplementary material: Interview guide for the physician and nurse involved in IPC

Supplementary material: Interview guide for the microbiologist

**Supplementary Table 1. Modifications to World Health Organization Infection Prevention and Control (IPC) Assessment Framework.**

| **Original question** | **Original response**  **(points)** | **Question change** | **New response options (points)** |
| --- | --- | --- | --- |
| Component 1:  4. Does the IPC team or focal person have dedicated time for IPC activities? | - No (0) - Yes (10) | ***(Reworded)***  4. How much dedicated time does the IPC team or focal person have for IPC activities? | - No time (0) - 1-<50% of the working day (2·5) - 50-<100% of working day (5) - 100% of the working day (10) |
| Component 1:  3. Does the IPC team have at least one full-time IPC professional  or equivalent (nurse or doctor working 100% in IPC) available? | - No IPC professional available (0) - No, only a part-time IPC professional available (2·5) - Yes, one per >250 beds (5) - Yes, one per ≤250 beds (10) | Same question | - No IPC professional available (0) - No, only a part-time IPC professional available (2.5) - Yes, one per **>** 110 beds (5) - Yes, one per **≤** 110 beds (10) |
| Component 6:  4. How frequently is the WHO Hand Hygiene Self-Assessment Framework Survey undertaken? ***(Removed)*** | - Never (0) - Periodically, but no regular schedule (2.5) - At least annually (5) | ***(New)***  4. How frequently do you monitor adherence to recommendations/guidelines for the following processes? | - Never or rarely (0) - Hand hygiene (at least annually) (1) - CLABSI prevention (at least annually) (1) - CAUTI prevention (at least annually) (1) - VAP prevention (at least annually) (1) - SSI prevention (at least annually) (1) - Appropriate use of PPE (at least annually) (1) - MDR transmission prevention (at least annually) (1) - *C. difficile* prevention (at least annually) (1) - Cleaning and disinfection (at least annually) (1) - Disinfection and sterilization (at least annually) (1) - Use of alcohol/hand rub (at least annually) (1) - Waste management (at least annually) (1) - Antimicrobial use (at least annually) (1) |
| Component 8: | NA | ***(Added)**** 3. Is drinking-water quality compliant with national regulations (e.g., residual chlorine 0·2 mg/L / 0·5 mg/L or 0 E. coli/100 mL and turbidity <5 NTU)? | - Water quality is not evaluated or is not compliant with national regulations (0) - Water quality is assessed at least annually, and it is compliant with national regulations (5) |
| Component 8:  6. Is functioning environmental ventilation (natural or mechanical) available inpatient care areas? | - No (0) - Yes (10) | Same question | - No (0) - Yes, there is functioning natural ventilation (2·5) - Yes, there is functioning natural and air-filtering system ventilation (positive for neutropenic/BMT patients and negative for airborne diseases) (5) |

*In order to maintain a maximum of 100 points earned per domain, we changed the score of questions 1 and 2 in component 8 so that the scores for each could be 0, 2·5, and 5 instead of 0, 2·5, and 7­·5. NTU: nephelometric turbidity units; CLABSI: central-line associated bloodstream infection; CAUTI: catheter-associated urinary tract infection; VAP: ventilator-associated pneumonia; SSI: surgical site infection; PPE: personal protective equipment, MDR: multi-drug resistant, *C. difficile*: *Clostridioides difficile*, BMT: bone marrow transplant.

**Supplementary Table 2. Complete answers to the Infection Prevention and Control Assessment Framework (IPCAF) survey by the 37 participating hospitals. Results are stratified by for-profit and non-for-profit hospitals.**

| **Question** | **For-profit**  n = 15 | **Non-profit**  n = 22 | **Overall**  n = 37 |
| --- | --- | --- | --- |
| **1. Infection Prevention Control (IPC) Programme** |  |  |  |
| 1.01 Does your facility have an IPC Programme? |  |  |  |
| No | 0 (0%) | 1 (5%) | 1 (3%) |
| Yes, without clearly defined responsibilities | 5 (33%) | 1 (5%) | 6 (16%) |
| Yes, with clearly defined responsibilities and annual work plan | 10 (67%) | 20 (91%) | 30 (81%) |
| 1.02 Do you have a team made up of IPC professionals? |  |  |  |
| No | 0 (0%) | 0 (0%) | 0 (0%) |
| No team, just one person is in charge of IPC | 1 (7%) | 2 (9%) | 3 (8%) |
| Yes, we have a team established | 14 (93%) | 20 (91%) | 34 (92%) |
| 1.03 Does the IPC team have at least one professional (doctor and/or nurse) in charge of IPC activities? |  |  |  |
| No IPC professional available | 0 (0%) | 0 (0%) | 0 (0%) |
| No, only one part-time IPC professional available | 0 (0%) | 0 (0%) | 0 (0%) |
| Yes, only one for > 110 beds | 6 (40%) | 5 (23%) | 11 (30%) |
| Yes, one for every 110 beds | 9 (60%) | 17 (77%) | 26 (70%) |
| 1.04 What is the experience of the professional (doctor and/or nurse) in charge of IPC activities? | | | |
| No experience | 0 (0%) | 1 (5%) | 1 (3%) |
| 1 to 5 years | 1 (7%) | 2 (9%) | 3 (8%) |
| > 5 years | 14 (93%) | 19 (86%) | 33 (89%) |
| 1.05 What is the time spent on specific activities by the IPC staff in relation to working hours? |  |  |  |
| 0 | 0 (0%) | 0 (0%) | 0 (0%) |
| Part-time (1 to 50% of the working day) | 1 (7%) | 3 (14%) | 4 (11%) |
| Part-time (>50% to <100% of the working day) | 3 (20%) | 2 (9%) | 5 (14%) |
| Full-time (100% of the working day) | 11 (73%) | 17 (77%) | 28 (76%) |
| 1.06 Does the IPC team include both doctors and nurses? |  |  |  |
| No | 0 (0%) | 0 (0%) | 0 (0%) |
| Yes | 15 (100%) | 22 (100%) | 37 (100%) |
| 1.07 Is there an IPC committee or equivalent actively supporting the IPC team (i.e. assisting in the development, implementation and monitoring of the programme)? |  |  |  |
| No | 0 (0%) | 5 (23%) | 5 (14%) |
| Yes | 15 (100%) | 17 (77%) | 32 (86%) |
| 1.08. Are any of the following professional groups represented/included in the IPC committee or its equivalent? |  |  |  |
| 1.08.01 Authorities of establishments (i.e. administrative director, general manager (executive director), medical director)? |  |  |  |
| No | 3 (20%) | 2 (9%) | 5 (14%) |
| Yes | 12 (80%) | 20 (91%) | 32 (86%) |
| 1.08.02 Senior clinical staff (i.e. doctor, nurse)? |  |  |  |
| No | 0 (0%) | 1 (5%) | 1 (3%) |
| Yes | 15 (100%) | 21 (95%) | 36 (97%) |
| 1.08.03 Facility management (i.e. biosecurity, waste and those responsible for water, sanitation and hygiene)? |  |  |  |
| No | 4 (27%) | 3 (9%) | 7 (19%) |
| Yes | 11 (73%) | 19 (86%) | 30 (81%) |
| 1.08.04 Quality and Patient Safety? |  |  |  |
| No | 0 (0%) | 11 (50%) | 11 (30%) |
| Yes | 15 (100%) | 11 (50%) | 26 (70%) |
| 1.09 Have you clearly defined the objectives of the IPC program? |  |  |  |
| No | 0 (0%) | 1 (5%) | 1 (3%) |
| Yes, only objectives of the IPC | 3 (20%) | 4 (18%) | 7 (19%) |
| Yes, IPC objectives and measurable outcome indicators | 3 (20%) | 5 (23%) | 8 (22%) |
| Yes, IPC objectives, measurable outcome indicators and defined of future objectives | 9 (60%) | 12 (55%) | 21 (57%) |
| 1.10 The senior authorities of the establishment demonstrate support and commitment to the IPC programme: |  |  |  |
| 1.10.1 Allocated budget for the IPC program (coverage of PCI activities, including salaries?) |  |  |  |
| No | 2 (13%) | 10 (45%) | 12 (32%) |
| Yes | 13 (87%) | 12 (55%) | 25 (68%) |
| 1.10.2 Demonstrable support for IPC objectives and their indicators within the establishment (i.e., at executive-level meetings, executive roundtables, participation in morbidity and mortality meetings)? |  |  |  |
| No | 3 (20%) | 6 (27%) | 9 (24%) |
| Yes | 12 (80%) | 16 (73%) | 28 (76%) |
| 1.11 Does your health facility have microbiological laboratory support (whether present or off-site) for routine purposes? |  |  |  |
| No | 0 (0%) | 0 (0%) | 0 (0%) |
| Yes, but the establishment is linked to a reference laboratory of another establishment=5 | 1 (7%) | 1 (5%) | 2 (5%) |
| Yes, and the results are delivered reliably (time and with sufficient quality) | 14 (93%) | 21 (95%) | 35 (95%) |
| **2. IPC Standards or Guidelines** |  |  |  |
| 2.01 Does your facility have experience in formulating or adapting standards or guidelines for IPC? |  |  |  |
| No | 0 (0%) | 1 (5%) | 1 (3%) |
| Yes | 15 (100%) | 21 (95%) | 36 (97%) |
| 2.02 Does your facility have guidelines available for: |  |  |  |
| 2.02.01 Standard precautions? |  |  |  |
| No | 0 (0%) | 2 (9%) | 2 (5%) |
| Yes | 15 (100%) | 20 (91%) | 35 (95%) |
| 2.02.02 Hand hygiene? |  |  |  |
| No | 0 (0%) | 1 (5%) | 1 (3%) |
| Yes | 15 (100%) | 21 (95%) | 36 (97%) |
| 2.02.03 Transmission-based precautions? |  |  |  |
| No | 0 (0%) | 2 (9%) | 2 (5%) |
| Yes | 15 (100%) | 20 (91%) | 35 (95%) |
| 2.02.04 Outbreak management and response preparedness? |  |  |  |
| No | 5 (33%) | 9 (41%) | 14 (38%) |
| Yes | 10 (67%) | 13 (59%) | 23 (62%) |
| 2.02.05 Prevention of surgical site infection? |  |  |  |
| No | 4 (27%) | 4 (18%) | 8 (22%) |
| Yes | 11 (73%) | 18 (82%) | 29 (78%) |
| 2.02.06 Prevention of catheter-associated vascular bloodstream infections? |  |  |  |
| No | 0 (0%) | 2 (9%) | 2 (5%) |
| Yes | 15 (100%) | 20 (91%) | 35 (95%) |
| 2.02.07 Prevention of hospital-acquired pneumonia (NAH); all types, including (but not limited to) pneumonias associated with mechanical ventilation? |  |  |  |
| No | 1 (7%) | 5 (23%) | 6 (16%) |
| Yes | 14 (93%) | 17 (77%) | 31 (84%) |
| 2.02.08 Prevention of catheter-associated urinary tract infections? |  |  |  |
| No | 1 (7%) | 5 (23%) | 6 (16%) |
| Yes | 14 (93%) | 17 (77%) | 31 (84%) |
| 2.02.09 Prevention of transmission of multidrug-resistant pathogens (MDRs)? |  |  |  |
| No | 3 (20%) | 5 (23%) | 8 (22%) |
| Yes | 12 (80%) | 17 (77%) | 29 (78%) |
| 2.02.10 Disinfection and sterilization? |  |  |  |
| No | 0 (0%) | 1 (5%) | 1 (3%) |
| Yes | 15 (15%) | 21 (95%) | 36 (97%) |
| 2.02.11 Occupational health according to national/international standards? |  |  |  |
| No | 2 (13%) | 9 (41%) | 11 (30%) |
| Yes | 13 (87%) | 13 (59%) | 26 (70%) |
| 2.02.12 Immunizations required for health personnel according to national/international standards? |  |  |  |
| No | 1 (7%) | 3 (14%) | 4 (11%) |
| Yes | 14 (93%) | 19 (86%) | 33 (89%) |
| 2.02.13 Safe injections? |  |  |  |
| No | 4 (27%) | 11 (50%) | 15 (41%) |
| Yes | 11 (73%) | 11 (50%) | 22 (59%) |
| 2.02.14 Waste management? |  |  |  |
| No | 0 (0%) | 0 (0%) | 0 (0%) |
| Yes | 15 (100%) | 22 (100%) | 37 (100%) |
| 2.02.15 Do you have activities integrated with ASP? |  |  |  |
| No | 1 (7%) | 5 (23%) | 6 (16%) |
| Yes | 14 (93%) | 17 (77%) | 31 (84%) |
| 2.03 Are your establishment's standards or guidelines based on the best available evidence and consistent with national/international standards (if any)? |  |  |  |
| No | 0 (0%) | 1 (5%) | 1 (3%) |
| Yes | 15 (100%) | 21 (95%) | 36 (97%) |
| 2.04 Is the implementation of the standards or guidelines adapted to local conditions? |  |  |  |
| No | 0 (0%) | 0 (0%) | 0 (0%) |
| Yes | 15 (100%) | 22 (100%) | 37 (100%) |
| 2.05 Is the direct patient care health team involved in planning and implementing IPC guidelines, in addition to the IPC team? |  |  |  |
| No | 4 (27%) | 6 (27%) | 10 (27%) |
| Yes | 11 (73%) | 16 (73%) | 27 (73%) |
| 2.06 Are relevant direct participants (i.e. lead doctors and nurses, hospital managers, quality management) involved in the formulation and adaptation of IPC guidelines, in addition to the team responsible for IPC? |  |  |  |
| No | 1 (7%) | 6 (27%) | 7 (19%) |
| Yes | 14 (93%) | 16 (73%) | 30 (81%) |
| 2.07 Does the health team receive specific training related to IPC guidelines each time they are released or updated? |  |  |  |
| No | 3 (20%) | 3 (14%) | 6 (16%) |
| Yes | 12 (80%) | 19 (86%) | 31 (84%) |
| 2.08 Does the institution have rules to reduce the risk of infections associated with buildings, repairs, remodeling and/or building demolitions? |  |  |  |
| No | 5 (33%) | 11 (50%) | 16 (43%) |
| Yes | 10 (67%) | 11 (50%) | 21 (57%) |
| **3. IPC Education and Training** |  |  |  |
| 3.01 Are there staff with experience in IPC to carry out IPC training? |  |  |  |
| No | 0 (0%) | 0 (0%) | 0 (0%) |
| Yes | 15 (100%) | 22 (100%) | 37 (100%) |
| 3.02 Are there additional staff not belonging to the IPC team with adequate skills to serve as trainers and monitors (i.e. nurses or liaison doctors, models among their peers)? Select an answer. |  |  |  |
| No | 2 (13%) | 9 (41%) | 11 (30%) |
| Yes | 13 (87%) | 13 (59%) | 26 (70%) |
| 3.03 How often are workers at the institution trained in basic IPC measures (i.e. hand hygiene, use of Personal Protective Equipment, Biosecurity)? Select an answer. |  |  |  |
| Never or rarely | 0 (0%) | 0 (0%) | 0 (0%) |
| Only at income (induction) | 0 (0%) | 5 (23%) | 5 (14%) |
| It is done upon admission and then at least one annual training (maintenance) in IPC for staff, but it is not mandatory | 7 (47%) | 11 (50%) | 18 (49%) |
| It is done upon admission and then at least one mandatory annual training (maintenance) in IPC for all staff | 8 (53%) | 6 (27%) | 14 (38%) |
| 3.04 How often are workers at the institution trained with respect to IPC-specific measures (i.e. packages of measures for the prevention of device-associated infections, prevention of surgical site infection, special isolation measures, detection and prevention of emergency and transmission of MMDR)? Select an answer. |  |  |  |
| Never or rarely | 0 (0%) | 3 (14%) | 3 (8%) |
| Only at income (induction) | 2 (13%) | 2 (9%) | 4 (11%) |
| It is done upon admission and then at least one annual training (maintenance) in IPC for staff, but it is not mandatory | 8 (53%) | 13 (59%) | 21 (57%) |
| It is done upon admission and then at least one mandatory annual training (maintenance) in IPC for all staff | 5 (33%) | 4 (18%) | 9 (24%) |
| 3.05 What topics related to IPC are included in the training of the institution's staff? |  |  |  |
| 3.05.01 Hand hygiene? |  |  |  |
| No | 0 (0%) | 0 (0%) | 0 (0%) |
| Yes | 15 (100%) | 22 (100%) | 37 (100%) |
| 3.05.02 Isolation measures and use of the PPE? |  |  |  |
| No | 0 (0%) | 0 (0%) | 0 (0%) |
| Yes | 15 (100%) | 22 (100%) | 37 (100%) |
| 3.05.03 Biosecurity (short-puncture accidents; immunization)? |  |  |  |
| No | 1 (7%) | 1 (5%) | 2 (5%) |
| Yes | 14 (93%) | 21 (95%) | 35 (95%) |
| 3.05.04 Packages of Measures to Prevent Device-Associated Infections? |  |  |  |
| No | 1 (7%) | 4 (18%) | 5 (8%) |
| Yes | 14 (93%) | 18 (82%) | 32 (86%) |
| 3.05.05 Prevention of surgical site infection? |  |  |  |
| No | 6 (40%) | 5 (23%) | 11 (30%) |
| Yes | 9 (60%) | 17 (77%) | 26 (70%) |
| 3.05.06 Detection and prevention of the emergence and transmission of MMDR (surveillance; Environmental cleaning and disinfection)? |  |  |  |
| No | 3 (20%) | 1 (5%) | 4 (11%) |
| Yes | 12 (80%) | 21 (95%) | 33 (89%) |
| 3.06 Do administrative and managerial staff receive general training regarding IPC in your health service? |  |  |  |
| No | 7 (47%) | 13 (59%) | 20 (54%) |
| Yes | 8 (53%) | 9 (41%) | 17 (46%) |
| 3.07 How are health workers and other staff members trained? Select an answer. |  |  |  |
| No training available | 0 (0%) | 1 (5%) | 1 (3%) |
| Written information or through oral instruction or only electronic learning | 6 (40%) | 7 (32%) | 13 (35%) |
| Additional interactive training sessions (includes simulation or in-service training) | 9 (60%) | 14 (64%) | 23 (62%) |
| 3.08 Are there regular evaluations regarding the effectiveness of training programmes (i.e. hand hygiene audits, use of Personal Protective Equipment, other knowledge checks)? Select an answer. |  |  |  |
| No | 1 (7%) | 2 (9%) | 3 (8%) |
| Yes, but not routinely | 6 (40%) | 9 (41%) | 15 (41%) |
| Yes, regularly (at least annually) | 8 (53%) | 11 (50%) | 19 (51%) |
| 3.09 Is IPC training integrated into clinical practice and training in other specialties (i.e., surgeon training includes aspects of IPC)? |  |  |  |
| No | 3 (20%) | 4 (18%) | 7 (19%) |
| Yes, in some disciplines | 11 (73%) | 14 (64%) | 25 (68%) |
| Yes, in all disciplines | 1 (7%) | 4 (18%) | 5 (14%) |
| 3.10 Is there IPC training tailored to patients or their families to minimize the potential for health care-acquired infections (i.e., immunosuppression patients, invasive device patients, patients with multidrug-resistant infections)? |  |  |  |
| No | 7 (47%) | 15 (68%) | 22 (59%) |
| Yes | 8 (53%) | 7 (32%) | 15 (41%) |
| 3.11 Are there continuing education opportunities for IPC staff (i.e. regular attendance at conferences, courses)? |  |  |  |
| No | 1 (7%) | 1 (5%) | 2 (5%) |
| Yes | 14 (93%) | 21 (95%) | 35 (95%) |
| **4. Surveillance of Health Care-Associated Infections (HAIs)** |  |  |  |
| 4.01.01 Is surveillance a well-defined component of your IPC program? |  |  |  |
| No | 0 (0%) | 0 (0%) | 0 (0%) |
| Yes | 15 (100%) | 22 (100%) | 37 (100%) |
| 4.01.02 Are there personnel responsible for surveillance activities? |  |  |  |
| No | 0 (0%) | 0 (0%) | 0 (0%) |
| Yes | 15 (100%) | 22 (100%) | 37 (100%) |
| 4.01.03 Have professionals responsible for surveillance activities been trained in basic epidemiology, surveillance and IPC(i.e. capacity to monitor surveillance methods and data management and interpretation)? |  |  |  |
| No | 1 (7%) | 1 (5%) | 2 (5%) |
| Yes | 14 (93%) | 21 (95%) | 35 (95%) |
| 4.01.04 Do you have IT support to carry out your surveillance (i.e. mobile technologies, electronic medical records, support from IT professionals)? |  |  |  |
| No | 1 (7%) | 5 (23%) | 6 (16%) |
| Yes | 14 (93%) | 17 (77%) | 31 (84%) |
| 4.02.01 Do you prioritize defining HAIs to be incorporated into surveillance according to the local context (i.e., identifying infections that are major causes of morbidity and mortality in the establishment)? |  |  |  |
| No | 0 (0%) | 1 (5%) | 1 (3%) |
| Yes | 15 (100%) | 21 (95%) | 36 (97%) |
| 4.02.02.a Surgical site infections? |  |  |  |
| No | 2 (13%) | 5 (23%) | 7 (19%) |
| Yes | 13 (87%) | 17 (77%) | 30 (81%) |
| 4.02.02.b Bacteremia associated with central vascular access? |  |  |  |
| No | 0 (0%) | 0 (0%) | 0 (0%) |
| Yes | 15 (100%) | 22 (100%) | 37 (100%) |
| 4.02.02.c Urinary catheter-associated urinary tract infection? |  |  |  |
| No | 0 (0%) | 0 (0%) | 0 (0%) |
| Yes | 15 (100%) | 22 (100%) | 37 (100%) |
| 4.02.02.d Pneumonia associated with mechanical ventilation? |  |  |  |
| No | 1 (7%) | 0 (0%) | 1 (3%) |
| Yes | 14 (93%) | 22 (100%) | 36 (97%) |
| 4.02.02.e *Clostridiodes difficile* infction? |  |  |  |
| No | 1 (7%) | 2 (9%) | 3 (8%) |
| Yes | 14 (93%) | 20 (91%) | 34 (92%) |
| 4.02.02.f Infections associated with central vascular access? |  |  |  |
| No | 1 (7%) | 0 (0%) | 1 (3%) |
| Yes | 14 (93%) | 22 (100%) | 36 (97%) |
| 4.02.02.g Clinically defined infections (i.e. definitions based on absence of microbiological testing)? |  |  |  |
| No | 5 (33%) | 3 (14%) | 8 (22%) |
| Yes | 10 (67%) | 19 (86%) | 29 (78%) |
| 4.02.02.h.01 MRSA |  |  |  |
| No | 2 (13%) | 5 (23%) | 7 (19%) |
| Yes | 13 (87%) | 17 (77%) | 30 (81%) |
| 4.02.02.h.02 VRE |  |  |  |
| No | 1 (7%) | 6 (27%) | 7 (19%) |
| Yes | 14 (93%) | 16 (73%) | 30 (81%) |
| 4.02.02.h.03 ESBL; Resistant to Carbapenem |  |  |  |
| No | 0 (0%) | 2 (9%) | 2 (5%) |
| Yes | 15 (100%) | 20 (91%) | 35 (95%) |
| 4.02.02.h.04 Carbapenem Resistant Non-fermenters |  |  |  |
| No | 0 (0%) | 3 (14%) | 3 (8%) |
| Yes | 15 (100%) | 19 (86%) | 34 (92%) |
| 4.02.02.h.05 *Candida auris* |  |  |  |
| No | 5 (33%) | 10 (45%) | 15 (41%) |
| Yes | 10 (67%) | 12 (55%) | 22 (59%) |
| 4.02.02.i Potentially epidemic infections (i.e. norovirus, influenza, tuberculosis, severe acute respiratory syndrome ('SARS'), Ebola, Lassa fever)? |  |  |  |
| No | 2 (13%) | 6 (27%) | 8 (22%) |
| Yes | 13 (87%) | 16 (73%) | 29 (78%) |
| 4.02.02.j in vulnerable populations (i.e. newborns, intensive care unit, immunocompromised, large burns)? |  |  |  |
| No | 3 (20%) | 1 (5%) | 4 (11%) |
| Yes | 12 (80%) | 21 (95%) | 33 (89%) |
| 4.02.02.k Infections that may affect health care workers in clinical, laboratory, or other settings (i.e., hepatitis B or C, human immunodeficiency virus (HIV), influenza, SARS-CoV2)? |  |  |  |
| No | 1 (7%) | 5 (23%) | 6 (16%) |
| Yes | 14 (93%) | 17 (77%) | 31 (84%) |
| 4.02.03 Do you regularly assess whether your surveillance system takes into account the needs and priorities of the moment? |  |  |  |
| No | 1 (7%) | 2 (9%) | 3 (8%) |
| Yes | 14 (93%) | 20 (91%) | 34 (92%) |
| 4.03 Surveillance methods |  |  |  |
| 4.03.01 Do you use standardized surveillance case definitions (numerator and denominator according to international definitions [i.e. NHSN-CDC/ECDC]) or if the definitions are adapted, has it been through a lengthy evidence-based process and expert consultation? |  |  |  |
| No | 1 (7%) | 0 (0%) | 1 (3%) |
| Yes | 14 (93%) | 22 (100%) | 36 (97%) |
| 4.03.02 Do you use standardized methods of data collection (i.e. prospective active surveillance) according to international surveillance protocols (i.e. NHSN-CDC/ECDC) or if adapted, through a lengthy evidence-based process and expert consultation? |  |  |  |
| No | 1 (7%) | 1 (5%) | 2 (5%) |
| Yes | 14 (93%) | 21 (95%) | 35 (95%) |
| 4.03.03 Do you have standardized processes in place to regularly review the quality of information (i.e., evaluation of case reporting formats, review of microbiology results, definition of denominators, etc.)? |  |  |  |
| No | 3 (20%) | 5 (23%) | 8 (22%) |
| Yes | 12 (80%) | 17 (77%) | 29 (78%) |
| 4.03.04 Do you have a Microbiology laboratory in your establishment with adequate capacity to support surveillance? Select an answer. |  |  |  |
| No | 0 (0%) | 0 (0%) | 0 (0%) |
| Yes, difference between gram-positive and negative strains, but does not identify pathogens | 0 (0%) | 0 (0%) | 0 (0%) |
| Yes, reliable identification of pathogens (identification by isolation) in a timely manner | 0 (0%) | 0 (0%) | 0 (0%) |
| Yes, reliable identification of pathogens and antimicrobial patterns (i.e., sensitivities) timely | 15 (100%) | 22 (100%) | 37 (100%) |
| 4.04.01 Is the information obtained from monitoring incorporated into the improvement plans adapted to the units/services in order to improve their IPC practices? |  |  |  |
| No | 1 (7%) | 0 (0%) | 1 (3%) |
| Yes | 14 (93%) | 22 (100%) | 36 (97%) |
| 4.04.02 Do you analyze antimicrobial resistance on a regular basis (i.e. quarterly/semi-annually/annually)? |  |  |  |
| No | 1 (7%) | 4 (18%) | 5 (14%) |
| Yes | 14 (93%) | 18 (82%) | 32 (86%) |
| 4.04.03 Do you regularly (for example, quarterly/half-yearly/annually) feedback up-to-date surveillance information to: |  |  |  |
| 4.04.03.a Frontline health care workers (doctors/nurses)? |  |  |  |
| No | 4 (27%) | 7 (32%) | 11 (30%) |
| Yes | 11 (73%) | 15 (68%) | 26 (70%) |
| 4.04.03.b Leaders/clinical directors of departments/services? |  |  |  |
| No | 1 (7%) | 5 (23%) | 6 (16%) |
| Yes | 14 (93%) | 17 (77%) | 31 (84%) |
| 4.04.03.c IPC Committee? |  |  |  |
| No | 0 (0%) | 3 (14%) | 3 (8%) |
| Yes | 15 (100%) | 19 (86%) | 34 (92%) |
| 4.04.03.d Non-clinical management/administration (Executive Manager, Financial Manager)? |  |  |  |
| No | 6 (40%) | 11 (50%) | 17 (46%) |
| Yes | 9 (60%) | 11 (50%) | 20 (54%) |
| 4.04.04 Does the institution report relevant surveillance data to sub-national and/or national authorities (i.e. HAIs, outbreaks, MMDR)? |  |  |  |
| Never or rarely | 2 (13%) | 6 (27%) | 8 (22%) |
| Regularly (at least annually) | 13 (87%) | 16 (73%) | 29 (78%) |
| 4.04.05 How is the feedback of the information provided by the surveillance carried out? (at least annually) |  |  |  |
| No feedback | 0 (0%) | 1 (5%) | 1 (3%) |
| Using written/oral information only | 3 (20%) | 10 (45%) | 13 (35%) |
| By presentation and finding problem-oriented solutions | 12 (80%) | 11 (50%) | 23 (62%) |
| **5. Multimodal Strategies for Implementation of IPC Interventions** | | | |
| 5.01 Do you use multimodal strategies to implement IPC interventions? |  |  |  |
| No | 0 (0%) | 4 (18%) | 4 (11%) |
| Yes | 15 (100%) | 18 (82%) | 33 (89%) |
| 5.02 Its multimodal strategies include some or all of the following elements: |  |  |  |
| 5.02.01 Changing systems? |  |  |  |
| Element not included in multimodal strategies | 0 (0%) | 4 (18%) | 4 (11%) |
| Interventions to ensure that the necessary infrastructure and continuous availability of supplies are available | 9 (60%) | 10 (45%) | 19 (51%) |
| Interventions to ensure the existence of the necessary infrastructure and the continuous availability of supplies addressing ergonomics and accessibility | 6 (40%) | 8 (36%) | 14 (38%) |
| 5.02.02 Education and training? |  |  |  |
| Element not included among the multimodal strategies | 1 (7%) | 3 (14%) | 4 (11%) |
| Written information or oral instruction or e-learning only | 4 (27%) | 8 (36%) | 12 (32%) |
| Additional interactive training sessions (includes simulation or in-service training) | 10 (67%) | 11 (50%) | 21 (57%) |
| 5.02.03 Monitoring and feedback? |  |  |  |
| Element not included in multimodal strategies | 0 (0%) | 3 (14%) | 3 (8%) |
| Monitoring compliance with processes or outcome indicators (i.e. hand hygiene audits) | 6 (40%) | 8 (36%) | 14 (38%) |
| Compliance monitoring and timely feedback with the results of monitoring to healthcare workers | 9 (60%) | 11 (50%) | 20 (54%) |
| 5.02.04 Communications and reminders? |  |  |  |
| Element not included in multimodal strategies | 0 (0%) | 4 (18%) | 4 (11%) |
| Reminders, posters, or other advocacy/awareness tools to promote intervention | 8 (53%) | 13 (59%) | 21 (57%) |
| Additional methods/initiatives to improve team communication between units and disciplines (i.e., when setting up case meetings and feedback rounds) | 7 (47%) | 5 (23%) | 12 (32%) |
| 5.02.05 Climate of security and cultural change? |  |  |  |
| Element not included in multimodal strategies | 1 (7%) | 12 (55%) | 13 (35%) |
| Managers/leaders show visible support and act as role models in promoting an adaptive approach to strengthen a culture that supports IPC and patient quality and safety | 7 (47%) | 6 (27%) | 13 (35%) |
| Additionally, as part of an adaptive approach to strengthen a culture that supports IPC quality of care and patient safety, teams and officials have been empowered by their interventions by stimulating a sense of belonging to them (i.e., participating in participatory rounds of information feedback) | 7 (47%) | 4 (18%) | 11 (30%) |
| 5.03 Is a multidisciplinary team involved in the implementation of multimodal IPC strategies? |  |  |  |
| No | 2 (13%) | 5 (23%) | 7 (19%) |
| Yes | 13 (87%) | 17 (77%) | 30 (81%) |
| 5.04 Do you regularly link patient safety and quality improvement colleagues to develop and promote multimodal IPC strategies? |  |  |  |
| No | 1 (7%) | 11 (50%) | 12 (32%) |
| Yes | 14 (93%) | 11 (50%) | 25 (68%) |
| 5.05 Do these strategies include bundles? |  |  |  |
| No | 0 (0%) | 4 (18%) | 4 (11%) |
| Yes | 15 (100%) | 18 (82%) | 33 (89%) |
| **6. Monitoring/Audit of IPC Practices and Feedback** | | | |
| 6.01 Do you have trained personnel responsible for monitoring/audit of IPC practices and feedback? |  |  |  |
| No | 1 (7%) | 2 (9%) | 3 (8%) |
| Yes | 14 (93%) | 20 (91%) | 34 (92%) |
| 6.02 Do you have a well-defined monitoring/audit plan with clear goals, objectives and activities (including tools to collect data systematically)? |  |  |  |
| No | 2 (13%) | 9 (41%) | 11 (30%) |
| Yes | 13 (87%) | 13 (59%) | 26 (70%) |
| 6.03 What preventive measures and how often are measurements of the level of adherence to them made? |  |  |  |
| 6.03.01 Hand hygiene (using the World Health Organization hand hygiene observation tool or equivalent)? |  |  |  |
| Less than once per year | 0 (0%) | 4 (18%) | 4 (11%) |
| Semi-annually to annually | 0 (0%) | 2 (9%) | 2 (5%) |
| At least quarterly | 15 (100%) | 16 (73%) | 31 (84%) |
| 6.03.02 Prevention of bacteremia associated with central vascular catheter? |  |  |  |
| Less than once per year | 1 (7%) | 5 (23%) | 6 (16%) |
| Semi-annually to annually | 0 (0%) | 2 (9%) | 2 (5%) |
| At least quarterly | 14 (93%) | 15 (68%) | 29 (78%) |
| 6.03.03 Prevention of urinary catheter-associated urinary tract infection? |  |  |  |
| Less than once per year | 2 (13%) | 5 (23%) | 7 (19%) |
| Semi-annually to annually | 0 (0%) | 2 (9%) | 2 (5%) |
| At least quarterly | 13 (87%) | 15 (68%) | 28 (76%) |
| 6.03.04 Prevention of pneumonia associated with mechanical ventilation? |  |  |  |
| Less than once per year | 2 (13%) | 5 (23%) | 7 (19%) |
| Semi-annually to annually | 2 (13%) | 2 (9%) | 4 (11%) |
| At least quarterly | 11 (73%) | 15 (68%) | 26 (70%) |
| 6.03.05 Prevention of surgical site infection? |  |  |  |
| Less than once per year | 2 (13%) | 10 (45%) | 12 (32%) |
| Semi-annually to annually | 3 (20%) | 4 (18%) | 7 (19%) |
| At least quarterly | 10 (67%) | 8 (36%) | 18 (49%) |
| 6.03.06 Proper Use of Personal Protective Equipment in Different Situations? |  |  |  |
| Less than once per year | 3 (20%) | 5 (23%) | 8 (22%) |
| Semi-annually to annually | 0 (0%) | 5 (23%) | 5 (14%) |
| At least quarterly | 12 (80%) | 12 (55%) | 24 (65%) |
| 6.03.07 Prevention of the spread of multidrug-resistant organisms (MDRs)? |  |  |  |
| Less than once per year | 2 (13%) | 5 (23%) | 7 (19%) |
| Semi-annually to annually | 3 (20%) | 2 (9%) | 5 (14%) |
| At least quarterly | 10 (67%) | 15 (68%) | 25 (68%) |
| 6.03.08 Prevention of C. difficile infections? |  |  |  |
| Less than once per year | 2 (13%) | 7 (32%) | 9 (24%) |
| Semi-annually to annually | 3 (20%) | 5 (23%) | 8 (22%) |
| At least quarterly | 10 (67%) | 10 (45%) | 20 (54%) |
| 6.03.09 Environmental cleaning and disinfection? |  |  |  |
| Less than once per year | 1 (7%) | 2 (9%) | 3 (8%) |
| Semi-annually to annually | 1 (7%) | 6 (27%) | 7 (19%) |
| At least quarterly | 13 (87%) | 14 (64%) | 27 (73%) |
| 6.03.10 Disinfection and sterilization of medical equipment/instruments? |  |  |  |
| Less than once per year | 6 (40%) | 5 (23%) | 11 (30%) |
| Semi-annually to annually | 1 (7%) | 3 (14%) | 4 (11%) |
| At least quarterly | 8 (53%) | 14 (64%) | 22 (59%) |
| 6.03.11 Consumption/use of alcohol solution for hand rubbing or soap? |  |  |  |
| Less than once per year | 5 (33%) | 7 (32%) | 12 (32%) |
| Semi-annually to annually | 1 (7%) | 4 (18%) | 5 (14%) |
| At least quarterly | 9 (60%) | 11 (50%) | 20 (54%) |
| 6.03.12 Waste management? |  |  |  |
| Less than once per year | 3 (20%) | 6 (27%) | 9 (24%) |
| Semi-annually to annually | 3 (20%) | 4 (18%) | 7 (19%) |
| At least quarterly | 9 (60%) | 12 (55%) | 21 (57%) |
| 6.03.13 Consumption/use of antimicrobial agents? |  |  |  |
| Less than once per year | 3 (20%) | 4 (18%) | 7 (19%) |
| Semi-annually to annually | 2 (13%) | 2 (9%) | 4 (11%) |
| At least quarterly | 10 (67%) | 16 (73%) | 26 (70%) |
| 6.04 Do you provide feedback to teams on the results of audits on the status of IPC activities? |  |  |  |
| 6.04.01 To the IPC team as part of the audit process? |  |  |  |
| No | 1 (7%) | 5 (23%) | 6 (16%) |
| Yes | 14 (93%) | 17 (77%) | 31 (84%) |
| 6.04.02 To heads of departments and managers of the audited areas? |  |  |  |
| No | 3 (20%) | 6 (27%) | 9 (24%) |
| Yes | 12 (80%) | 16 (73%) | 28 (76%) |
| 6.04.03 To frontline health care workers from across the clinical service? |  |  |  |
| No | 5 (33%) | 12 (55%) | 17 (46%) |
| Yes | 10 (67%) | 10 (45%) | 20 (54%) |
| 6.04.04 To the IPC committee or quality of care committees or equivalent? |  |  |  |
| No | 1 (7%) | 7 (32%) | 8 (22%) |
| Yes | 14 (93%) | 15 (68%) | 29 (78%) |
| 6.04.05 To hospital administration and higher authorities? |  |  |  |
| No | 5 (33%) | 7 (32%) | 12 (32%) |
| Yes | 10 (67%) | 15 (68%) | 25 (68%) |
| 6.05 Is the data in the monitoring reports regularly reviewed (at least twice a year)? |  |  |  |
| No | 1 (7%) | 3 (14%) | 4 (11%) |
| Yes | 14 (93%) | 19 (86%) | 33 (89%) |
| 6.06 Are the monitoring and feedback of IPC processes and indicators carried out under a "non-punitive" institutional culture oriented to improvement and behavioral changes? |  |  |  |
| No | 0 (0%) | 2 (9%) | 2 (5%) |
| Yes | 15 (100%) | 20 (91%) | 35 (95%) |
| 6.07 Do you evaluate cultural safety factors in your facility (i.e. when using other surveys such as HSOPSC, SAQ, PSCHO, HSC22)? |  |  |  |
| No | 10 (67%) | 19 (86%) | 29 (78%) |
| Yes | 5 (33%) | 3 (14%) | 8 (22%) |
| **7. Workload, Staffing and Bed Occupancy** |  |  |  |
| 7.01 Staffing |  |  |  |
| 7.01.01 Does the institution assess staffing according to patient workload using a standard assessment or staffing needs assessment tool? |  |  |  |
| No | 8 (53%) | 11 (50%) | 19 (51%) |
| Yes | 7 (47%) | 11 (50%) | 18 (49%) |
| 7.01.02 Does the institution maintain an adequate workload of health care workers according to the number of patients through the facility? |  |  |  |
| No | 1 (7%) | 8 (36%) | 9 (24%) |
| Yes, for staff in less than 50% units | 2 (13%) | 6 (27%) | 8 (22%) |
| Yes, for staff in more than 50% of units | 6 (40%) | 3 (14%) | 9 (24%) |
| Yes, for all health care workers in the facility | 6 (40%) | 5 (23%) | 11 (30%) |
| 7.01.03 Do you have a system in place to act on the results of staffing needs assessments when the establishment plan is considered to be too low? |  |  |  |
| No | 6 (40%) | 14 (64%) | 20 (54%) |
| Yes, for staff in less than 50% units | 0 (0%) | 0 (0%) | 0 (0%) |
| Yes, for staff in more than 50% of units | 0 (0%) | 0 (0%) | 0 (0%) |
| Yes, for all health care workers in the facility | 9 (60%) | 8 (36%) | 17 (46%) |
| 7.02 Bed occupancy |  |  |  |
| 7.02.01 Are you considering the design of your room in accordance with international standards for hospital bed capacity? |  |  |  |
| No | 1 (7%) | 8 (36%) | 9 (24%) |
| Yes, but only in certain departments | 8 (53%) | 11 (50%) | 19 (51%) |
| Yes, for all units (including emergency departments and pediatrics) | 6 (40%) | 3 (14%) | 9 (24%) |
| 7.02.02 Is bed occupancy maintained in your facility to one patient per bed? |  |  |  |
| No | 0 (0%) | 1 (5%) | 1 (3%) |
| Yes, but only in certain departments | 4 (27%) | 7 (32%) | 11 (30%) |
| Yes, for all units (including emergency departments and pediatrics) | 11 (73%) | 14 (64%) | 25 (68%) |
| 7.02.03 Do you place patients in beds in the hallway outside the room (including beds in the emergency department)? |  |  |  |
| Yes, more often than twice a week | 1 (7%) | 8 (36%) | 9 (24%) |
| Yes, less often than twice a week | 2 (13%) | 2 (9%) | 4 (11%) |
| No | 12 (80%) | 12 (55%) | 24 (65%) |
| 7.02.04 You ensure adequate distancing of > 1 m between beds |  |  |  |
| No | 0 (0%) | 1 (5%) | 1 (3%) |
| Yes, but only in certain departments | 5 (33%) | 12 (55%) | 17 (46%) |
| Yes, for all departments (including emergency department and pediatrics) | 10 (67%) | 9 (41%) | 19 (51%) |
| 7.02.05 Do you have a system to identify a high occupancy rate in hospital beds, which will help you adopt alternative solutions? |  |  |  |
| No | 0 (0%) | 2 (9%) | 2 (5%) |
| Yes, it is the responsibility of the department head | 2 (13%) | 8 (36%) | 10 (27%) |
| Yes, it is the responsibility of hospital administration/management | 13 (87%) | 12 (55%) | 25 (68%) |
| **8. Built Environment, Materials and Equipment for IPC at Hospital Level** | | | |
| 8.01 Water |  |  |  |
| 8.01.01 Are water services available at all times and in sufficient quantity for all uses (i.e. hand washing, drinking, personal hygiene, medical activities, sterilization, decontamination, cleaning and laundry)? |  |  |  |
| No, available on average < 5 days per week | 0 (0%) | 0 (0%) | 0 (0%) |
| Yes, available on average > 5 days per week or every day but not in sufficient quantity | 1 (7%) | 0 (0%) | 1 (3%) |
| Yes, every day and in sufficient quantity | 14 (93%) | 22 (100%) | 36 (97%) |
| 8.01.02 Is there a source of drinking water that is accessible to health personnel, patients and families at all times and in all locations/wards? |  |  |  |
| No, not available | 0 (0%) | 5 (23%) | 5 (14%) |
| Sometimes, either only in some places or not available to all users | 0 (0%) | 7 (32%) | 7 (19%) |
| Yes, accessible at all times and for all rooms/ groups | 15 (100%) | 10 (45%) | 25 (68%) |
| 8.01.03 Is the quality of the water supplied in the institution periodically monitored in accordance with local regulations (i.e. heavy metals: concentration below maximum permitted levels, residual chlorine concentration: 0.2 mg/L / 0.5 mg/L, E. coli concentration: 0 E. coli/100 mL and turbidity <5 NTU (nephelometric turbidity units)? |  |  |  |
| No, it is not evaluated or the recommended levels of quality parameters are not reached | 0 (0%) | 4 (18%) | 4 (11%) |
| Yes, it is evaluated at least once a year and the water has appropriate levels | 15 (100%) | 18 (82%) | 33 (89%) |
| 8.02 Hand hygiene and sanitary facilities |  |  |  |
| 8.02.01 Are hand hygiene devices (i.e. alcohol-based solution for hand rubbing or soap and water with handwashing sinks and clean single-use towels) working and available in all care locations? |  |  |  |
| No, devices are not present | 0 (0%) | 0 (0%) | 0 (0%) |
| Yes, devices are available but only in some areas | 0 (0%) | 2 (9%) | 2 (5%) |
| Yes, devices are in all areas, but supplies (inputs) are not reliably available | 2 (13%) | 8 (36%) | 10 (27%) |
| Yes, reliably available in all areas including entrance and waiting rooms | 13 (87%) | 12 (55%) | 25 (68%) |
| 8.02.02 At your facility, are at least 4 or more improved toilets or latrines available for outpatients or at least 1 per 20 inpatient users? |  |  |  |
| Less than 50% of the required number of latrines available and in operation | 1 (7%) | 4 (18%) | 5 (14%) |
| Present in sufficient number but not all functioning | 0 (0%) | 6 (27%) | 6 (16%) |
| Present in sufficient number and functioning | 14 (93%) | 12 (55%) | 26 (70%) |
| 8.03 Energy source, ventilation and cleaning |  |  |  |
| 8.03.01 Is there sufficient energy/energy source available in your health care facility in the day and night for all uses (i.e. pumping and boiled water, sterilization and decontamination, incineration or alternative treatment technologies, electronic medical devices, general lighting of areas where health care procedures are performed, to ensure safe health care and lighting of toilets and showers)? |  |  |  |
| Yes, always but only in some of the areas mentioned | 3 (20%) | 4 (18%) | 7 (19%) |
| Yes, always and in all areas mentioned | 12 (80%) | 18 (82%) | 30 (81%) |
| 8.03.02 Is operating ambient ventilation (natural or based on mechanical or filtered air systems) available in the patient's general care areas? |  |  |  |
| No | 0 (0%) | 2 (9%) | 2 (5%) |
| Yes, there is only natural ventilation | 0 (0%) | 6 (27%) | 6 (16%) |
| Yes, there is a mechanical ventilation system | 3 (20%) | 6 (27%) | 9 (24%) |
| Yes, there is a filtered air system (hePA filters with positive pressure for neutropenic/bone marrow transplant patients; negative pressure for aerosol-borne diseases) | 12 (80%) | 8 (36%) | 20 (54%) |
| 8.03.03 For horizontal floors and work surfaces, is there a record of the cleaning carried out and signed by the staff responsible for cleaning on a daily basis? |  |  |  |
| There are no records of cleaning floors and surfaces | 2 (13%) | 6 (27%) | 8 (22%) |
| The record exists, but is not filled daily or is out of date | 2 (13%) | 8 (36%) | 10 (27%) |
| Yes | 11 (73%) | 8 (36%) | 19 (51%) |
| 8.03.04 Are appropriate materials available in good condition for cleaning (i.e. detergent, mops, buckets, and others)? |  |  |  |
| No materials available | 0 (0%) | 1 (5%) | 1 (3%) |
| Yes, there are materials available, but not well maintained | 3 (20%) | 9 (41%) | 12 (32%) |
| Yes, there are materials available and well maintained | 12 (80%) | 12 (55%) | 24 (65%) |
| 8.04 Placing Patients in Healthcare Settings and Personal Protective Equipment |  |  |  |
| 8.04.01 Do you have individual rooms with negative pressure in case of aerosol airborne transmission (i.e. tuberculosis, measles) or for cohort in case of multidrug-resistant germ infections if the number of individual rooms for isolation is insufficient? |  |  |  |
| No | 3 (20%) | 7 (32%) | 10 (27%) |
| There are no single rooms, but there are rooms suitable for cohort of patients | 1 (7%) | 3 (14%) | 4 (11%) |
| Yes, there are single rooms | 11 (73%) | 12 (55%) | 23 (62%) |
| 8.04.02 Is PPE always available, adequate and in sufficient quantities for all health workers? |  |  |  |
| No | 0 (0%) | 0 (0%) | 0 (0%) |
| Yes, but not continuously available in sufficient quantities and of poor quality | 2 (13%) | 5 (23%) | 7 (19%) |
| Yes, continuously available in sufficient quantities and of adequate quality | 13 (87%) | 17 (77%) | 30 (81%) |
| 8.05 Medical waste and wastewater management |  |  |  |
| 8.05.01 Do you have functional containers for the collection of non-infectious (general) waste, infectious waste and short-sharp waste, close to all waste generation points? |  |  |  |
| They do not have containers or segregated elimination of short-sharps | 0 (0%) | 0 (0%) | 0 (0%) |
| There are separate containers, but no lids or they are filled to more than 3/4 of their capacity; only two containers (instead of three); or only at some waste generation points | 0 (0%) | 4 (18%) | 4 (11%) |
| Yes, there are suitable containers for waste collection | 15 (100%) | 18 (82%) | 33 (89%) |
| 8.05.02 Is there a functional fenced waste pit/landfill or municipal landfill available for non-infectious (non-hazardous/general) waste disposal? |  |  |  |
| No, there are no pits | 3 (20%) | 7 (32%) | 10 (27%) |
| There is a pit/landfill in the facility but insufficient dimensions; overfilled or unfenced/closed wells/landfills; or irregular collection of municipal waste | 0 (0%) | 1 (5%) | 1 (3%) |
| Yes, is there a functional burial well/fenced waste dump or municipal collection available for the disposal of non-infectious (non-hazardous/general) waste? | 12 (80%) | 14 (64%) | 26 (70%) |
| 8.05.03 Is there a functional and sufficiently capable incinerator or other alternative treatment technology for the treatment of infectious and cutting waste (i.e. an autoclave)? |  |  |  |
| No, none present | 5 (33%) | 10 (45%) | 15 (41%) |
| Present, but not functional | 2 (13%) | 0 (0%) | 2 (5%) |
| Yes, there is a system for the treatment of infectious and cutting waste | 8 (53%) | 12 (55%) | 20 (54%) |
| 8.05.04 Is there safe handling of wastewater with on-site treatment (i.e. septic tank followed by drainage pit) or sent to a functioning sewer system? |  |  |  |
| No, not present | 1 (7%) | 4 (18%) | 5 (14%) |
| Present, but not in operation | 1 (7%) | 1 (5%) | 2 (5%) |
| Yes, there is a safe wastewater treatment system | 13 (87%) | 17 (77%) | 30 (81%) |
| 8.06 Decontamination and sterilization |  |  |  |
| 8.06.01 Does your health care facility provide a dedicated decontamination area or does it have a sterile supply department for the decontamination and sterilization of medical devices and other supplies/equipment? |  |  |  |
| No, not present | 0 (0%) | 1 (5%) | 1 (3%) |
| Present, but not in operation | 0 (0%) | 0 (0%) | 0 (0%) |
| Yes, there is a sterilization center | 15 (100%) | 21 (95%) | 36 (0%) |
| 8.06.02 Do you have sterile and disinfected equipment, reliably, ready for use? |  |  |  |
| No, available on average less than five days per week | 0 (0%) | 1 (5%) | 1 (3%) |
| Yes, available on average more than five days per week or every day, but not in sufficient quantity | 1 (7%) | 5 (23%) | 6 (16%) |
| Yes, available every day and in sufficient quantity | 14 (93%) | 16 (73%) | 30 (81%) |
| 8.06.03 Are disposable items available when needed? (i.e., safe injection devices, examination gloves)? |  |  |  |
| No, not available | 0 (0%) | 1 (5%) | 1 (3%) |
| Yes, but only sometimes available | 0 (0%) | 2 (9%) | 2 (5%) |
| Yes, available continuously | 15 (100%) | 19 (86%) | 34 (92%) |

**Interview guide for IPC physicians or nurses**.

1. Are you aware of how long the IPC has been operating within your hospital?

a. If they responded that the program is new, further explore into why it took so long to implement the program.

1. Who is part of the program?
2. What are the goals of the IPC program at your institution?
3. If COVID is mentioned in their response, ask them to describe other goals besides COVID and/or if these goals have changed since COVID.
4. If the answer includes bigger goals, such as the implementation of chlorhexidine baths, inquire about how they decide on the goals they should work on. Example, in what units is surveillance performed? Which units/health care workers do they go to for the implementation of prevention packets?
5. What are the main barriers that prevent the IPC from achieving these goals?
6. What could the IPC do to achieve these goals?
7. Can you describe the support from hospital leadership towards the IPC?
8. Is there any collaboration between the Ministry of Health or local/regional government health organizations and the IPC?
9. Can you describe what this relationship looks like?
10. How would you describe the work relationship between the IPC team and other health care workers/ hospital workers?

(Inquire about each):

- 1. Physicians, including internists and surgeons
  2. Nurses
  3. Microbiologists
  4. Cleaning staff
  5. Sterilization and disinfection personnel
  6. Administration/Legal
  7. Regional/ national health authorities

1. As an infection control professional, how would you describe your ability to communicate with medical staff?
2. What are some things that have helped improve communication in the workplace? Ask for examples of good communication.
3. How would you describe the hospital staff’s adherence to the IPC measurements set at your hospital?
4. Why would you describe it this way?
5. What actions does the IPC take if there is low adherence to hand washing, or a high rate of HAIs?
6. Does the hospital uses any incentives (financial or non-financial) to reduce the rate of HAIs or infections caused by multidrug-resistant organisms?
7. What do you think motivates people to change their behavior with regards to IPC activities at your hospital?
8. Are the HAIs rates and level of adherence to hand hygiene routinely communicated with units/services in the hospital?
9. How would you describe your ability to implement interventions within your hospital?
10. What are some of the main barriers that prevented this?
11. What helped make the implementation successful?
12. How would you describe your ability to involve medical staff in infection prevention initiatives at your hospital?

- *Prompts: What are some of the things that make it difficult to involve the medical staff? What has been successful?*

1. Can mention any intervention(s) that the IPC has implemented in recent years that was successful?
   1. What was the intervention?
   2. How was success measured?
   3. What do you think was key in achieving the success of the intervention (example, there was a key person)?
2. Can you think of an intervention that was unable to be implemented or that failed after implementation?
3. Please explain why you think the intervention failed and what would be necessary to successfully implement it next time.

- *Prompts:*

1. *Please describe how human resources and salary influence IPC activities at your hospital.*
2. *Please describe how microbiology laboratory resources influence IPC activities at your hospital.*
3. *Please describe how access to technology resources influences IPC activities at your hospital. (example, to track data and provide feedback on this data to implement changes for improvement.)*
4. *Please describe how the infrastructure of medical facilities and medical supplies influence IPC activities at your hospital.*
5. *Please describe how hospital management/administration influences IPC activities at your hospital.*
6. *Please describe how IPC education and training influences IPC activities at your hospital.*
7. *Please describe how the support or commitment of medical staff influences IPC activities at your hospital.*
8. *Please describe how nursing support influences IPC at your hospital.*
9. *Please describe how the work environment influences IPC activities at your hospital.*

**Interview guide for microbiologists.**

1. What is your role as a microbiologist in IPC?
2. How would you describe your collaboration with IPC?
3. Is there an interaction with Ministry of Health or local government health organizations? Could you describe what this relationship is like? And how does it benefit the lab?
4. Do you have the resources to properly identify microorganisms and their resistance mechanisms?
5. In general, how often do they run out of supplies for microbiological diagnostics?
   - How do they solve it?
6. Can you briefly describe the process of communicating the results of multidrug-resistant organisms (MDROs) in clinical samples?
7. How long does it take for microbiological test results to be reported in a reasonable amount of time? (e.g., swabs for detection of multidrug-resistant organisms)?

- And this is 24/7?
- Does this process differ for surveillance swab results, if any?

1. Do they have the capacity to investigate outbreaks of multidrug-resistant organisms? (it can be to determine if they are the same strain, it can be environmental samples)
2. Can you describe any strategies implemented to detect colonization with MDROSs:

- Methicillin-resistant staphylococcus
- Vancomycin-resistant enterococcus
- ESBL Enterobacteriaceae (Extended Spectrum Beta-Lactamase Producers)
- Carbapenem-resistant Enterobacteriaceae

1. Is *Clostridioides difficile* testing available at your HCF?
2. What could the microbiology lab do to help the IPC?
3. What are the main barriers that the microbiology laboratory faces to support IPC in your HCF?
